# Efficient Reconstruction of Predictive Consensus Metabolic Network Models

**DOI:** 10.1371/journal.pcbi.1005085

**Published:** 2016-08-26

**Authors:** Ruben G. A. van Heck, Mathias Ganter, Vitor A. P. Martins dos Santos, Joerg Stelling

**Affiliations:** 1 Department of Biosystems Science and Engineering and Swiss Institute of Bioinformatics, ETH Zurich, Basel, Switzerland; 2 Laboratory of Systems and Synthetic Biology, Wageningen University, Wageningen, The Netherlands; 3 LifeGlimmer GmbH, Berlin, Germany; University of Wisconsin-Madison, UNITED STATES

## Abstract

Understanding cellular function requires accurate, comprehensive representations of metabolism. Genome-scale, constraint-based metabolic models (GSMs) provide such representations, but their usability is often hampered by inconsistencies at various levels, in particular for concurrent models. COMMGEN, our tool for COnsensus Metabolic Model GENeration, automatically identifies inconsistencies between concurrent models and semi-automatically resolves them, thereby contributing to consolidate knowledge of metabolic function. Tests of COMMGEN for four organisms showed that automatically generated consensus models were predictive and that they substantially increased coherence of knowledge representation. COMMGEN ought to be particularly useful for complex scenarios in which manual curation does not scale, such as for eukaryotic organisms, microbial communities, and host-pathogen interactions.

## Author Summary

Many large-scale mathematical models describe metabolism to understand how microbes and other organisms (including humans) function and interact with each other and with their environment. Making these models is extremely time- and effort-intensive; it requires gathering and combining information from many sources, including the organism’s genome sequence, biological databases, scientific literature, and expert advice. The exact procedure and resources used depend on the model creators’ expertise and research interests, such that independently created models for the same organism are often very different and can hardly be compared. However, each model typically contains unique information that is ‘lost’ when working with a different model. To integrate the available knowledge, we developed a computational tool to build consensus metabolic models. Our tool—COMMGEN- combines independently generated models by matching identical parts and resolving differences between inconsistent parts. We apply our tool to four sets of models of different organisms. In all these sets, COMMGEN identified and resolved hundreds of inconsistencies. COMMGEN can be generally applied to standardize and improve models of metabolism, in particular for complex scenarios, such as those involving microbial communities and host-pathogen interactions.

## Introduction

Genome-scale constraint-based metabolic models (GSMs) are curated organism-specific knowledge repositories [1]. They integrate many distinct (bio)chemical entities and typically account for thousands of metabolites, reactions and genes. When assuming that metabolism is in a steady state, GSMs also enable metabolic simulations with applications in genome annotation [2,3], analysis of omics data [4–6], phenotype predictions [7–9], organism comparison [9–12], drug discovery [7,13,14], and metabolic engineering [8,15]. GSMs thereby quantitatively reconstruct the internal metabolic and transport wiring of the modeled organism and thus increase our systems level understanding.

Genome-scale metabolic reconstructions consist of metabolites, metabolic reactions (including boundary reactions and a biomass reaction), cellular compartments, and genes [1,16]. The reactions are organized according to the cellular compartments in which they are active. Enzyme-driven (as opposed to spontaneous) reactions are associated with Gene-protein-reaction rules (GPR), which include one or more genes. For multiple genes, the GPR indicates whether alternative isozymes or enzyme complexes catalyze the reaction [17]. A reaction’s equation consists of substrates and products with their corresponding stoichiometries. A reaction’s reversibility describes whether the reaction operates forward, backward, or bi-directionally. The reaction flux bounds specify the reaction’s capacity, that is, the absolute upper and lower bounds of the reaction flux. Transport reactions transfer metabolites between cellular compartments, whereas boundary reactions define nutrient uptake and secretion. The biomass reaction, finally, reflects the molecular composition of a cell or organism and represents cell or organism growth. Together, these entities and their encoding in a GSM aim to represent the current knowledge of the organism’s metabolism.

However, even for well-studied organisms such as *Saccharomyces cerevisiae* or *Bacillus subtilis*, many uncertainties remain during GSM construction. These uncertainties are typically manually addressed based on expert knowledge and scientific literature, which involves a laborious iterative process that can take several years, for example, for eukaryotes [1]. The main sources of uncertainties are: (i) incomplete and erroneous information from heterogeneous and potentially contradictory data sources such as insufficiently curated and inconsistent gene annotations [18], alternative naming and spelling variants of metabolites (different namespaces) [18–21], and conflicting reaction reversibilities [2,22]; (ii) subjectivity in interpreting literature sources; (iii) integration of qualitative and quantitative data (e.g., inconsistent growth data); and (iv) incompatible levels of detail between and among (reference) databases; for example, databases may represent metabolic pathways by detailed individual reactions or by a single lumped reaction [18], and they may use varying structural definitions for metabolite classes such as lipids and polymers [21,23].

As a consequence, when several GSMs for the same organism are developed independently, they are complementary and only partially overlapping [24,25]. The extent of variation between models for the same organism can be dramatic. For example, the well-established human and yeast GSMs agree only on 3% [18] and 35% [20] of their reactions, respectively, when ignoring electron, proton, and water imbalances. Differences between GSMs resulting from different modeling frameworks and model authors can even be more substantial than biological differences between organisms [26]. Any GSM-driven analysis, which needs to (somewhat arbitrarily) select one GSM when several are available, thus, only operates on a subset of the available information.

To represent metabolism more comprehensively, and thereby improve our understanding of a target organism, alternative GSMs of a target organism can be integrated into a so-called consensus model of the respective organism, one per organism. Consensus models have an increased scope (by combining unique parts of initial GSMs) and they are more consolidated (by identifying shared parts of initial GSMs that are likely to be reliable). When discrepancies exist between GSMs, these must be carefully examined to select the most appropriate modeling alternative. However, while consensus models have been generated successfully for several (model) organisms such as budding yeast and human, this required extensive manual curation by communities of domain experts [10,20,24,25,27]. To alleviate this bottleneck and render GSMs truly useful for the understanding of cellular function and evolution, community function, and host-pathogen interactions, semi-automatic consensus model generation approaches have been proposed. It has been shown that the combination of complementary GSMs of the same organism reduces existing gaps in individually reconstructed GSMs [28,29]. These approaches focused mainly on reconciling namespaces (a particularly important challenge for matching metabolites) or on curating the underlying databases [18,21]. Thereby, existing methods address only a small subset of the problems in consensus model generation described above. For example, they do not identify and curate cases when two initial GSMs represent the same metabolic process at different levels of granularity [30].

Here, we present COMMGEN, a tool for COnsensus Metabolic Model GENeration that reconciles two or more distinct GSMs of the same organism beyond a common namespace. COMMGEN automatically identifies similarities, dissimilarities, and complements of the metabolic networks based on an extensive classification of problems that typically arise during GSM integration and on novel algorithms to resolve these problem classes. For several model organisms, we show that semi-automatically created consensus GSMs in a standardized namespace [31] are substantially more consolidated than achievable by a common namespace alone, and that they retain or even improve on the initial GSMs’ predictive capabilities. Because the consensus GSMs contain the information from each initial GSM, they comprehensively represent our best understanding of the organisms’ metabolic networks.

## Results

Our analyses addressed model building, testing and refinement in a stepwise fashion. We started by identifying the classes of inconsistencies that exist between models for four widely different albeit representative microbes. We subsequently set up the framework for COnsensus Metabolic Model GENeration, and tested it on the four case studies for functionality and predictability.

### Inconsistency classes arising in model merging

**Fig 1:**
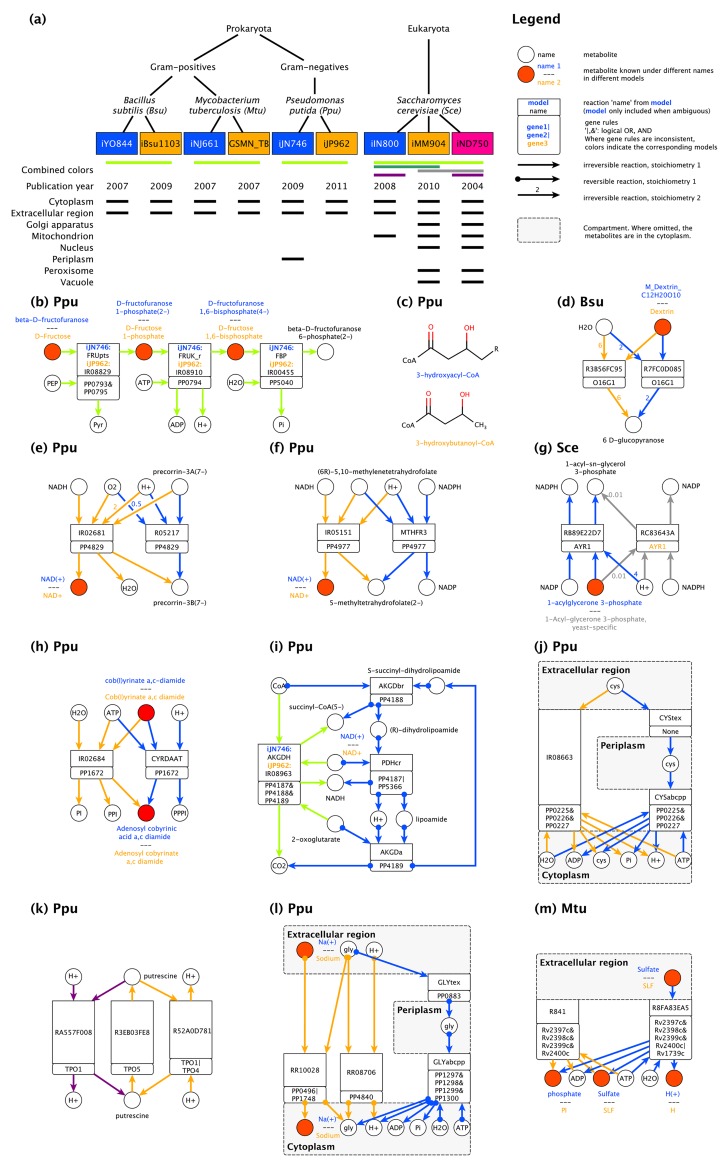
Models used in this study and classification of inconsistencies. (**a**) Overview of the used initial GSMs. (**b**) Instances of identical metabolites with different MnXRef identifiers. (**c**) Non-identical metabolites that perform identical functions in the network context. (**d**) Alternative modeling of polymers. (**e**) Nested and encompassing reactions. (**f**) Alternative usage of redox pairs. (**g**) Alternative reactions with consequences for redox metabolism. (**h**) Partially overlapping reactions differing in phosphate products. (**i**) Lumped vs. non-lumped representation of a pathway. (**j**) Invalid transport reaction (IR08663). (**k**) Alternative transport reactions for putrescine. (**l**) Alternative transport reactions for glycine. (**m**) Invalid boundary reaction (R841). Circles represent chemical species, arrows chemical reactions, and grey boxes different compartments. Red nodes indicate instances of identical species within the network context whose alternative names are separated by horizontal lines. Rectangular boxes contain the original reaction names, rounded rectangles their corresponding GPRs, where '&' represents a logical AND, and '|' a logical OR. Edges with filled circles represent reversible reactions. Stoichiometric coefficients unequal to one are indicated at their respective arrows. The shown reactions originate from GSMs of four different organisms: *B*. *subtilis* (**d**), as represented in iYO844 [3] (blue) and iBSu1103 [36] (orange); *M*. *tuberculosis* (**m**), as represented in iNJ661 [14] (blue) and GSMN_TB [7] (orange);*P*. *putida* (**b,c,e,f,h,I,j,k**), as represented in iJN746 [33] (blue) and iJP962 [10] (orange); and *S*. *cerevisiae* (**g,l**), as represented in iIN800 [48] (blue) and iMM904 [18,37] (orange) and iND750 [49] (pink).

To systematically resolve inconsistencies between two or more Initial GSMs (IGSMs) to be integrated, we defined three main (coupled) inconsistency categories: metabolites, reactions, and compartments. We explain these categories and the inconsistency classes they contain using examples from four sets of IGSMs that cover gram-positive and gram-negative bacteria as well as yeast (Fig 1a).

#### Metabolites

IGMs often represent a specific chemical compound differently because metabolite identifiers are ambiguous and they reside in different namespaces [31]. When one simply merges IGSMs, that is, adds the IGSMs’ contents, this leads to redundant pathways (Fig 1b) that may differ in metabolites, gene associations, stoichiometries, and reversibilities. The essential step of identifying and merging different metabolites that represent the same chemical compound in different namespaces has been emphasized previously [29–31]. However, more complicated situations exist when different metabolites actually represent different chemical compounds, but these compounds have the same function in their network context. This typically arises when metabolites are modeled at different granularity, for example, as ‘iron’ and ‘Fe^2+^’, or ‘glucose’ and ‘alpha-D-glucose’. Common metabolites may also have different chemical sum formulas in different IGSMs, for example, depending on whether functional groups are specified or not (Fig 1c), or when polymers are modeled with a different numbers of subunits (Fig 1d). In such cases, the merging of metabolites has to prevent stoichiometric inconsistencies in the consensus model: if a merged polymer can be produced from fewer subunits than result from its degradation, mass conservation is violated. Hence, a common namespace is not sufficient to identify common metabolites in IGSMs.

#### Reactions

A particular biological process is often represented differently in two models because of uncertainties, disagreements, errors, and modeling decisions, resulting in alternative representations of a single reaction or of reaction sets. These alternatives need to be identified and matched to avoid reaction redundancies (Fig 1b) and violations of mass balances due to inconsistent stoichiometries (Fig 1c and 1d). However, inconsistencies may extend beyond namespaces and stoichiometries. They often result from modeling decisions, both in capturing individual reactions, and in the granularity of representation for metabolic processes. Nested reactions, where one reaction is a perfect subset of another reaction with respect to metabolites, are possible consequences. In the example in Fig 1e, the cofactor NADH may be used, but it is not required—for a consensus model, a decision between these alternatives eventually has to be made. Alternative modeling decisions on cofactor usage are common in IGSMs as shown in Fig 1f with a ‘choice’ between using NADH and NADPH and in Fig 1g, where the same chemical conversion can either yield NADP from NADPH or NADPH from NADP. More complex cases to resolve are partially overlapping reactions and lumped reactions, where multiple reactions are artificially represented by fewer reactions. Fig 1h shows an example of two alternative reactions that generate triphosphate or pyrophosphate and monophosphate, respectively; simply merging the two IGSMs would feed the side-products into different pathways because no reaction exists that interconverts these metabolites directly. Such inconsistencies are not only found between IGSMs, where they are expected, but also within IGSMs, as demonstrated in Fig 1i. Hence, it is important to consider the network context of the IGSMs and of the merged GSM.

#### Compartments

IGSMs of the same organism may consider different subcellular compartments (Fig 1a), affecting the localization and multiplicity of reactions as well as the incorporated transport reactions. For example, in Fig 1j, the two IGSMs for a gram-negative bacterium have the same net reaction for the import of cysteine into the cytoplasm. In one IGSM this requires one reaction because the periplasm is not explicitly modeled, whereas the more detailed transport in the other IGSM requires two reactions. After identifying this class of inconsistencies, a consensus model can either replace the transporter connecting the extracellular space with the cytoplasm by two reactions, or remove the entire periplasm and retain a single transport reaction. Because transporters and transport reactions are notoriously difficult to identify and characterize [1], IGSMs are often inconsistent in transport reactions. Fig 1k shows an extreme example: a single merging artifact effectively destroys the model of the proton gradient because protons can be transported across the membrane in either direction by simultaneous import and export of putrescine. Inconsistencies in transport reactions can also lead to thermodynamically infeasible cycles [1] such as ATP generation resulting from cycling glycine over the membrane (Fig 1l). Finally, boundary reactions, which are not mass-balanced because they exchange material with the environment, are sometimes lumped with transport reactions for the same chemical compound and thus first require standardization (Fig 1m). Overall, therefore, a broad spectrum of unrelated but interconnected inconsistencies at the metabolite, reaction, and compartment levels need to be identified and resolved for consensus model generation.

### The COMMGEN framework

**Fig 2:**
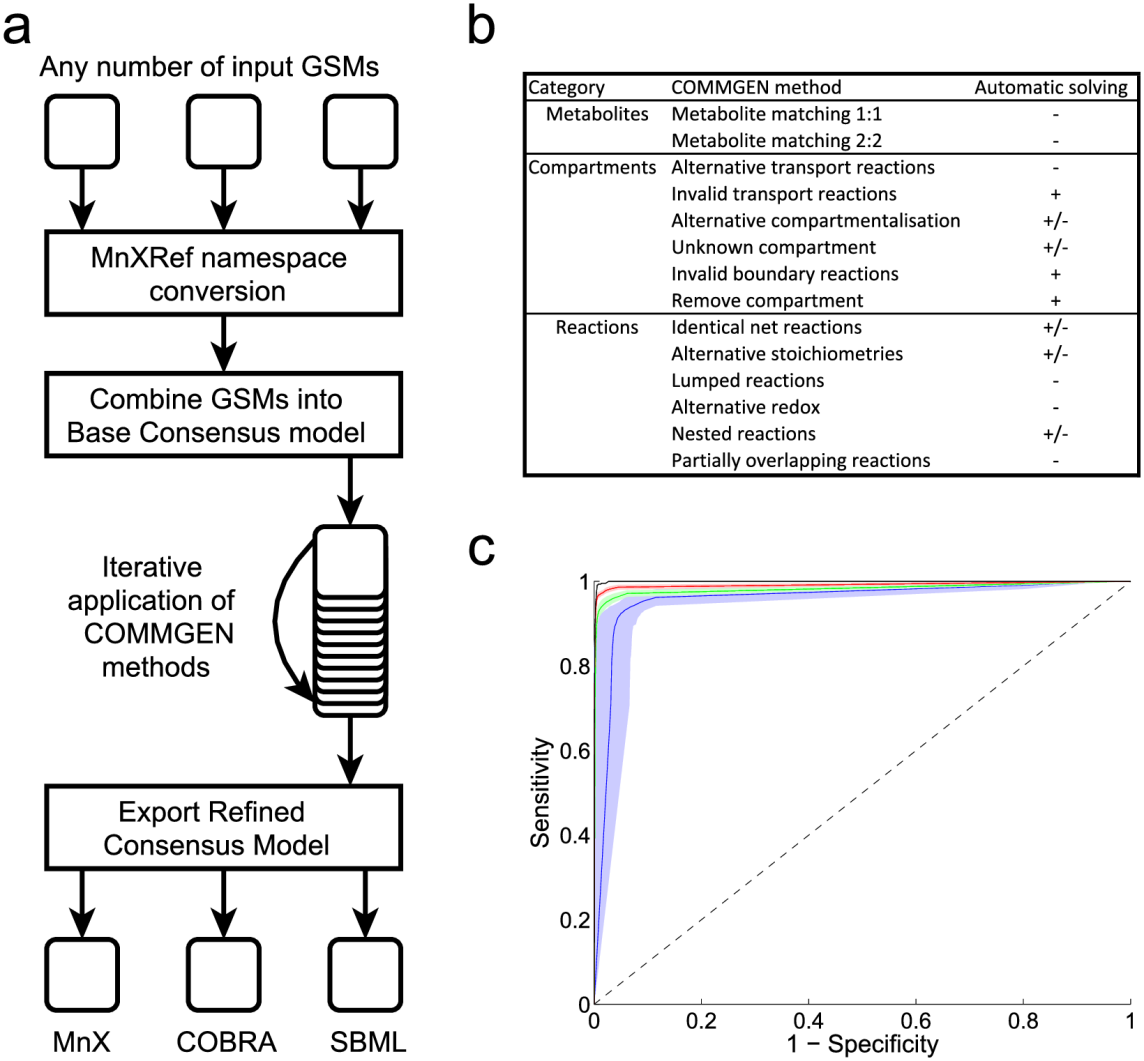
COMMGEN framework. (**a,b**) Overview of COMMGEN workflow and available methods. The COMMGEN methods are either fully automatic (+), conditionally or optionally automatic (+/-), or they always require manual intervention (-). (**c**) Performance of the metabolite matching methods if run without manual intervention, leading to ROC-curves of the classification of metabolites as identical or non-identical based on their network context. Lines correspond to different fractions of the network information being randomly discarded: black, 0%; red, 30%; green, 60%; blue, 90%. The shades indicate the standard deviations in the classification. The data presented here was obtained using the *Pseudomonas putida* GSMs iJP962 [10] and iJN746 [33]; analysis results for the other sets of GSMs and additional information can be found in S5 Protocol.

COMMGEN is a software tool that is designed to address the above problems in consensus model generation, leading to a semi-automatic reconciliation of two or more GSMs for a given organism. In terms of software architecture, COMMGEN operates on GSMs in SBML format [32], the standard modeling language for systems biology (Fig 2a). The IGSMs are first converted into a common chemical naming system using the MnXRef namespace [31]. Next, COMMGEN combines all reactions of the IGSMs into a Basic Consensus Model (BCM). The BCM is used to identify and reconcile inconsistencies between and within the IGSMs, ultimately yielding a Refined Consensus Model (RCM) in SBML format. Because many inconsistencies are interconnected, it is difficult to identify a consensus between IGSMs, to distinguish between conflicting and complementary model parts, and to resolve all inconsistencies automatically. COMMGEN therefore resolves all unambiguous cases automatically, and it guides the user to decide on the remaining cases. COMMGEN records all changes such that the user can automatically repeat the procedure with minimal effort, including manual alterations of previously made choices.

To identify and address all the different inconsistency classes described above, COMMGEN iteratively applies a set of independent methods (Fig 2b). All methods automatically identify instances of their respective inconsistency classes. Metabolite matching is a core element of model merging. We developed a novel algorithm to identify sets of metabolites that represent the same chemical compound based on their network context, that is, their neighboring metabolites and reactions, thereby addressing the issue of different granularity in IGSMs for metabolites (see Methods for details). Performance tests for *P*. *putida* networks revealed very high sensitivity and specificity of the algorithm, even when only a minority of the network is used to infer matching metabolite sets (Fig 2c). Metabolite matching allows COMMGEN subsequently to reconcile the associated reactions: metabolites are merged, through which novel pathways and branching points can be formed, and alternative representations of biochemical reactions become apparent. Specifically, COMMGEN matches sets of reactions in the following categories (see Methods for the respective algorithms): (i) reactions with identical metabolites but different stoichiometries; (ii) nested reactions; (iii) reactions that differ only in redox pairs; (iv) partially overlapping reactions; and (v) lumped reactions. Furthermore, it deals with differences in subcellular compartmentalization by (i) facilitating the removal of transporters; (ii) enabling the removal of entire compartments; (iii) resolving differences in the modeling of boundary reactions; (iv) identifying different transport reactions for the same metabolite across the same membrane; and (v) identifying identical biochemical conversions in different compartments.

COMMGEN’s methods differ in the extent to which identified inconsistencies can be resolved automatically (Fig 2b). For some categories, the user can choose to automatically handle inconsistencies, for example, to deal with differences in reaction directionality. Conditionally automatic refers to inconsistency classes where some instances can be addressed automatically, but others cannot: if two matched reactions differ only in stoichiometric coefficients, COMMGEN can automatically select the elementally balanced reaction, but only when exactly one reaction is balanced. Manual intervention is always possible, and it is required when inconsistencies are too complex and diverse for a well-performing heuristic for automation. Manual curation is also advisable when an erroneous choice may substantially impact model performance. For example, a single incorrect match between two metabolites with different chemical sum formulas can have severe consequences for the correctness of model predictions. Hence, although the COMMGEN method for network-based metabolite matching performs extremely well (Fig 2c), we recommend manual confirmation of predicted matches.

### Model generation with COMMGEN: Case study for *P*. *putida*

To describe COMMGEN operation in detail and to evaluate the framework’s performance, we focus on consensus model generation for *Pseudomonas putida*, for which the two GSMs iJP962 [8,10] and iJN746 [33] have been developed independently (Fig 1a). The initial overlap between these two models is surprisingly low: they only have 58% of their genes, 33% of their metabolites and 2% of their reactions in common. Conversion into the MnXRef namespace [31] only increases the common part to 44% for metabolites and 11% for reactions.

**Fig 3:**
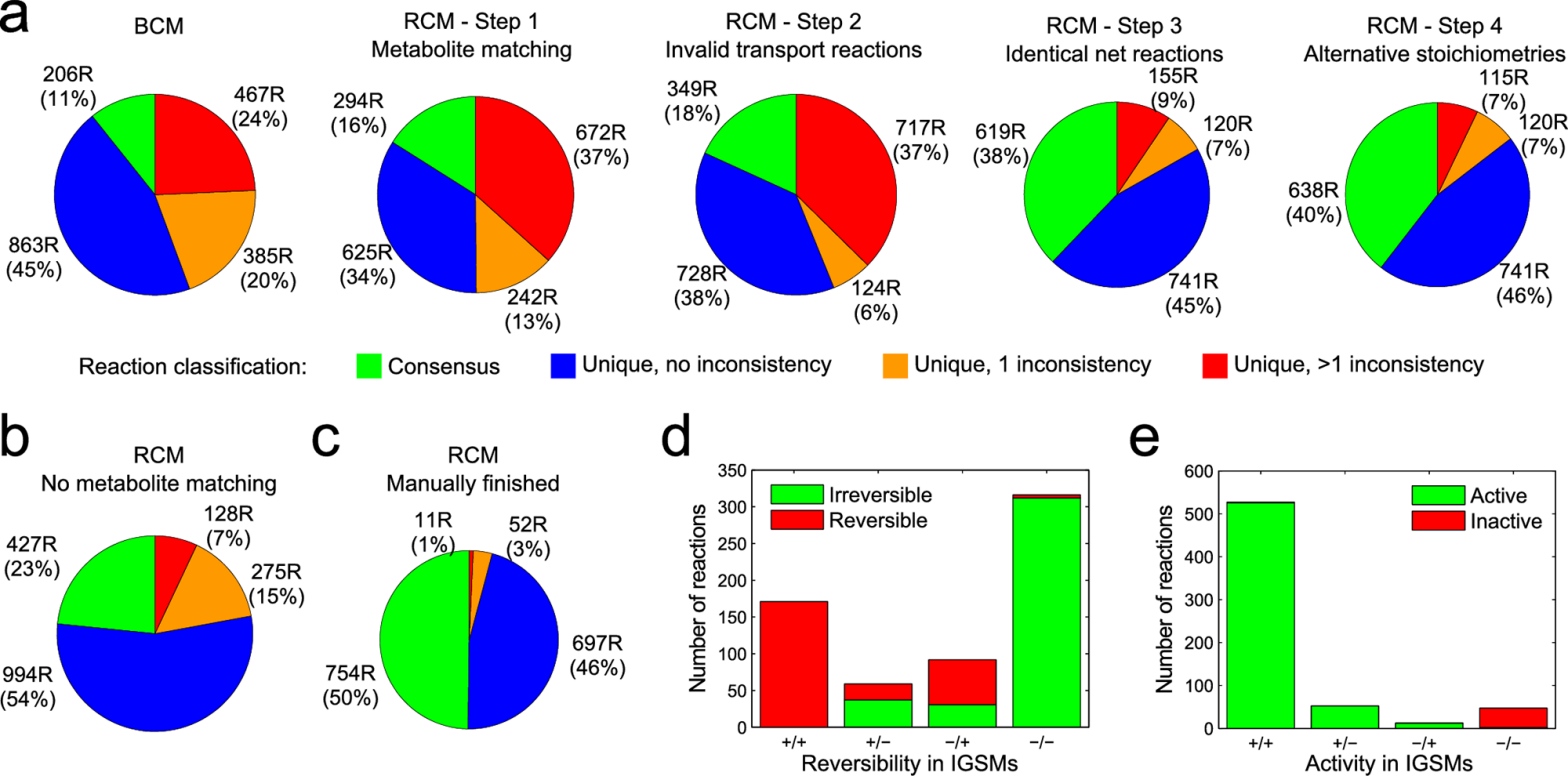
Application of COMMGEN to *P*. *putida* GSMs. (**a**) Automatic inconsistency identification and reconciliation substantially increases consensus and reduces inconsistencies. Reactions are classified into consensus reactions (green) and unique reactions involving no (blue), a single (orange), or multiple (red) inconsistencies. (**b, c**) Characteristics of the refined consensus model as in (**a**) without network-based metabolite matching (**b**), or after manually addressing the remaining inconsistencies (**c**). (**d**) Numbers of reversible (‘+’) and irreversible (‘-‘) reactions in the RCM, grouped by the four possible combinations of reversibilities in the IGSMs. (**e**) Numbers of active and inactive reactions in the RCM, grouped by being active (‘+’) or inactive (‘-‘) in the IGSMs.

To quantitatively determine the occurrences of inconsistencies and their resolution, we classify reactions as consensus reactions (shared between the GSMs) and unique reactions. We further categorize unique reactions according to whether they are unrelated to any inconsistency, related to a single inconsistency, or related to multiple inconsistencies (a reaction may appear in the last category because COMMGEN methods are not mutually exclusive in the inconsistencies they identify). Because the identified inconsistencies ultimately depend on namespace consistency, user-defined settings, and user choices, we quantified the resolution of inconsistencies by automatic processing to remove user bias as much as possible. After creating the BCM from the IGSMs and merging the identical reactions, the fraction of consensus reactions was low (11%) and approximately half of the unique reactions were associated with at least one inconsistency (Fig 3a; S1 Protocol). The inconsistencies exemplified in Fig 1 are, thus, not isolated cases; they merely illustrate the main problems in consensus model generation.

Next, we employed a four-step automatic process to reconcile inconsistencies between the IGSMs and to converge to an automatically generated RCM (Fig 3a). First, COMMGEN increased the namespace consistency through our network context-based metabolite matching method (note that we manually confirmed the proposed matches such that subsequently identified inconsistencies were not overestimated). This increased the overlap to 53% for metabolites and 16% for reactions. In the second step, COMMGEN addressed the difference in cellular compartments in the *P*. *putida* GSMs (Fig 1a). In particular, transport reactions from iJP962 that immediately take up metabolites from the extracellular space into the cytoplasm were split such that they match the transport processes from iJN746, and periplasmic instances of the involved metabolites were added. Next, COMMGEN identified and merged sets of reactions with practically (ignoring protons and water) identical net formula. These sets include reactions that have different GPR rules or different reaction directionalities, or that did not have identical net formulas prior to the splitting of transport reactions or the COMMGEN-based metabolite matching. In this step, we processed inconsistent reaction reversibilities using our previously published method to predict reaction directionalities based on metabolite patterns [2], and we processed inconsistent gene associations by combining the GPR rules with a ‘strict’ heuristic (see S2 Protocol). Finally, COMMGEN identified and merged reactions that involve the same metabolites, but differ in stoichiometric coefficients; directionality and GPR inconsistencies were handled as above.

The detailed data shown in Fig 3a emphasize the interdependencies of inconsistencies that may arise in model merging, in particular, that resolving inconsistencies may facilitate subsequent identification of more inconsistencies, resulting in an increased number of identified inconsistent reactions. The four automated steps increased the share of reactions that are consensus reactions originating from both IGSMs from 11% (in the BCM) to 39% (in the RCM), while also substantially reducing the number of reactions associated with inconsistencies (Fig 3a). We evaluated the significance of the metabolite matching step by re-running the process without it, which lead to only 23% consensus reactions (Fig 3b). In addition, we used the automatically generated RCM as the starting point for manual curation guided by COMMGEN methods. This allowed us to reconcile most of the remaining inconsistencies and to obtain a consensus for 50% of the reactions (Fig 3c). In summary, our detailed case study for *P*. *putida* therefore provides evidence for the efficiency of the COMMGEN framework, and in particular of its novel methods such as network context-based metabolite matching.

### Automatically generated consensus models are functional and predictive

**Fig 4:**
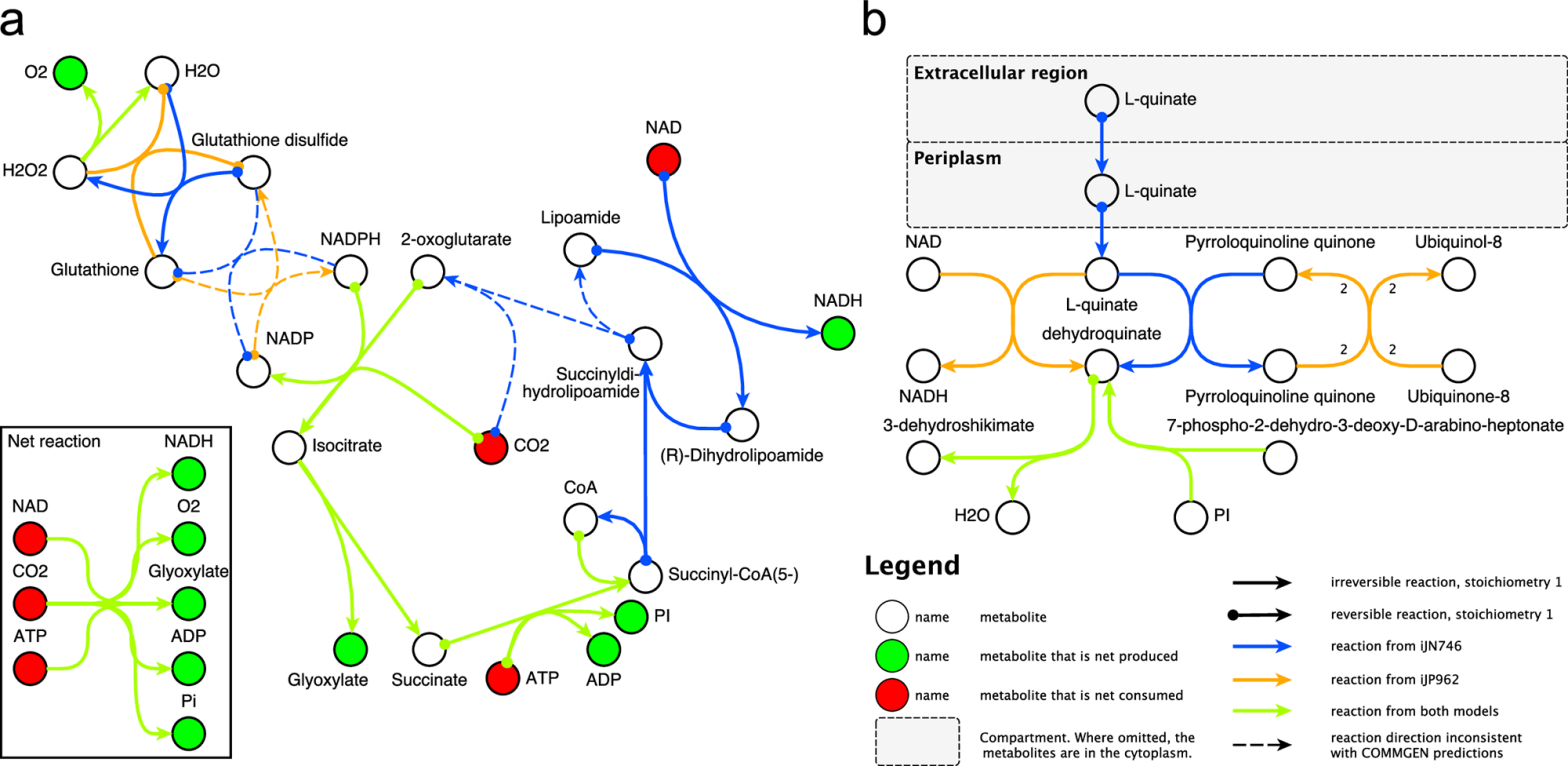
Subnetwork analysis for *P*. *putida*. (**a**) Example error of ‘naïve’ iGSM merging where the initial *P*. *putida* BCM contains a biologically inaccurate carbon dioxide fixation cycle due to incorrect directionalities in the IGSMs. This error is automatically resolved as COMMGEN assigns reaction directionalities opposite to those shown with dashed reaction arrows. (**b**) Example for a new metabolic function in the consensus model. *P*. *putida* can grow on L-quinate as its sole carbon source. Neither of the initial models captures this behavior, whereas the consensus model provides the necessary, complementary reactions.

We next asked, to what extent automated consensus model generation preserved or even extended functionality of the IGSMs, initially focusing on the *P*. *putida* models. Our automated method involved the probabilistic prediction of reaction directionalities [2] to resolve reaction inconsistencies, instead of simply setting all reactions with conflicting directionalities to reversible, which would tend to overestimate the organism’s metabolic capabilities. It maintained reaction directions in case of consensus between the IGSMs, although the prediction method is agnostic to matches between models; it constrained directions in many cases when such constraints existed in only one IGSM (Fig 3d). The benefits of this approach are best exemplified with a concrete example (Fig 4a). The *P*. *putida* BCM contains a small set of reactions that together allow for non-physiological CO_2_ fixation. This incorrect CO_2_ fixation cycle was automatically removed when inconsistent directionalities of a reaction present in both IGSMs were processed, thereby preventing a major error in the RCM. Note that direction prediction also identified a reaction assigned with a direction that is not consistent with the remainder of the network (see also Fig 1i), namely a directed lumped reaction common to both IGSMs, and a bidirectional non-lumped reaction set present in only one model. Another important aspect of model consolidation is the extent to which active reactions in the IGSMs (that is, reactions that can carry metabolic flux in principle) are preserved. As shown in Fig 3e, essentially all active reactions in one of the networks remained active in the RCM, and only reactions that were non-functional in both IGSMs remained inactive. In growth phenotype predictions, the RCM occasionally disagreed with all IGSMs, suggesting ‘new’ metabolic functions. For example, while neither of the IGSMs captured that *P*. *putida* can grow on L-quinate as sole carbon source, complementation of reactions in the RCM enabled a biologically consistent model behavior (Fig 4b). These aspects together indicate overall functionality of the automatically generated consensus model.

**Fig 5:**
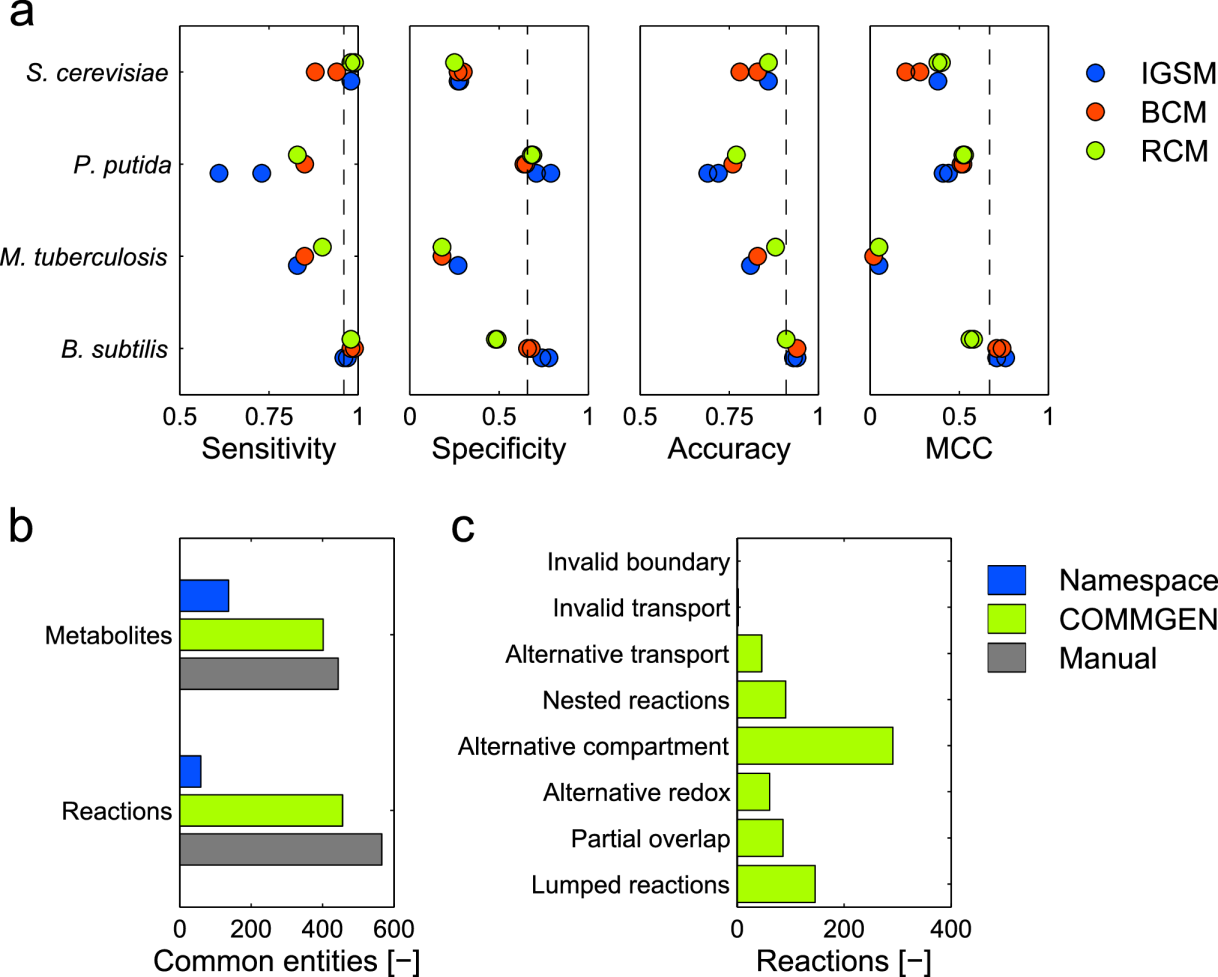
Performance evaluation of COMMGEN. (**a**) Evaluation of GSM ability to predict growth phenotypes. Predictive ability of initial GSMs (blue), basic consensus models (red), and automatically created refined consensus model (green) according to the metrics defined in the text. The test data comprised gene knockout data (*B*. *subtilis* [3,36], *P*. *putida* [8,50], *M*. *tuberculosis* [51], *S*. *cerevisiae* [49]), biolog data (*B*. *subtilis* [3,36], *P*. *putida* [8,33]) and auxotrophies (*P*. *putida* [50]). See S3 Protocol for details. (**b,c**) Comparison of manual (yeast consensus model [20] based on the IGSMs iMM904 [37] and iLL672 [38]) and automatic consensus model generation with namespace matching only, or with COMMGEN. (**b**) Numbers of common reactions and metabolites for manual curation, name space conversion, and automatically created refined consensus model. (**c**) Incidences of inconsistent reaction classes identified by COMMGEN.

The performance of GSMs as mathematical models for cellular metabolism is typically evaluated by assessing their ability to correctly predict wild type and mutant growth phenotypes across different growth conditions [34]. We performed corresponding simulations for automatically refined consensus models as well as for their ancestors (IGSMs and BCM) for each of the four evaluated organisms (Fig 1a). Specifically, we computed sensitivity, specificity, accuracy, and Matthew’s correlation coefficient (MCC; unlike accuracy it takes the total numbers of true and false test cases into account) [35] for growth phenotype predictions (see S3 Protocol for details). Fig 5a shows the performance indicators for the IGSMs, the BCMs, and the automatically refined consensus models for each organism. In nearly all metrics, the IGSMs outperformed the BCM (except for *P*. *putida*), and they were outperformed by the RCM (except for *B*. *subtilis*). For *B*. *subtilis*, resolving inconsistencies in the BCM decreased all scores except sensitivity. This can be explained by one IGSM (iBSu1103) being largely based on a predecessor (iYO844); in addition, iBSu1103 was optimized for correct growth predictions using GrowMatch [34,36]. Information from iYO844 can thus include errors that were deliberately removed from iBSu1103 and it can reverse changes made by the performance optimization. Thus, although the prediction profiles of the RCMs largely resemble the IGSM profiles, RCMs on average outperform both the IGSMs and the BCMs, indicating efficiency of the automated consensus model generation methods in COMMGEN even in terms of prediction capabilities. Notably, user choices of the biomass reaction do not influence the performance substantially (Fig 5a), pointing to robustness of the methods as well.

### Automatic reconciliation is comparable to manual consensus model generation

Finally, we wanted to evaluate how automatic consensus model generation compares to its (largely) manual counterpart. We focused on the community approach to establish a yeast consensus model [20] based on the IGSMs iMM904 [37] and iLL672 [38] because this first model reconciliation effort is especially well documented. Fig 5b shows that transfer of the IGSMs into a standardized namespace alone identifies only small subsets of common metabolites and reactions. COMMGEN’s automated reconciliation method, in contrast, achieves nearly the same extent of matching between the IGSMs as reported for the manual curation. The automatically generated RCM showed good performance in mutant phenotype predictions (sensitivity = 0.98, specificity = 0.28, accuracy = 0.87 and MCC = 0.42; note that a comparison to the manual consensus model is impossible because the community effort did not aim at establishing a model suitable for FBA). In addition, COMMGEN directly identifies many inconsistencies between model reactions that result, for example, from different numbers of compartments in the IGSMs (Fig 5c). These would be clear starting points for domain experts for subsequent COMMGEN-assisted manual curation. We believe that the combination of automated procedures with close-to-manual quality and of support for targeted manual curations would substantially enhance future community efforts.

## Discussion

Genome-scale constraint-based metabolic models are both integrated knowledge repositories and predictive mathematical models. In terms of knowledge representation, a consensus model should be more consolidated than individual GSMs due to shared parts, more comprehensive due to unique parts, and more accurate due to reconciliation of inconsistencies in similar parts. A consensus model, however, can propagate errors in the initial models’ unique parts, and it may be less consistent than the initial models, especially when inconsistencies in similar model parts were not identified or reconciled.

Inconsistencies in GSMs are typically nested, not mutually exclusive, and therefore difficult to address, which so far prevented the development of methods for the automated generation of consensus models [30]. Manual network reconciliation, the predominant approach applied today, is difficult and cumbersome because the number of inconsistencies between just two or three IGSMs already runs in the thousands. Based on a systematic classification of inconsistencies, COMMGEN automatically identifies and semi-automatically reconciles inconsistencies between and within two or more IGSMs. The inconsistencies could theoretically be reconciled fully automatically, but automated resolution depends on the used reference databases, which vary to a large extent [18]. Therefore, COMMGEN does not entirely remove the need for manual inspection and curation. For example, our framework relies on network similarity between alternative realizations of metabolites and reactions in order to match them. Because the reactions surrounding biomass formation are often implemented very differently in different GSMs, they are not matched. While our implementation lets the user choose one of the IGSM biomass reactions, a manual update seems necessary as long as COMMGEN does not automatically fetch external information that would enable an automatic reconciliation of the biomass reaction. In addition, there exists a trade-off between sensitivity and specificity for the identification of inconsistent reactions, which limits the detection of lumped and non-lumped pathway representations with a different net reaction. Also, the identification of similar or identical reactions in different cellular compartments is difficult to achieve automatically (but an extension of the current framework could progress in this direction by combining the information from metabolite instances in different compartments prior to metabolite matching). COMMGEN thus forms a necessary bridge between full automation and high-quality manual curation for consensus metabolic model generation.

Regarding a GSM’s predictive mathematical model character, it is important to note that remaining inconsistencies in a consensus model can have severe effects, for example, when inconsistencies resulting from model merging are not adequately addressed. As a consequence, individual GSMs may outperform a consensus model in terms of predictive ability even though the latter is more representative of the available information. COMMGEN’s aim (and design) is to compare and reconcile IGSMs in order to obtain a high-quality representation of the IGSMs’ combined information. In contrast to model optimization methods such as GrowMatch [34], COMMGEN does not create a model optimized for predictive ability, and it does not use corresponding experimental information. However, our example applications also demonstrated that automatically generated consensus models almost always have higher predictive power than the manually curated IGSMs and that these models can be comparable to manually constructed consensus models as shown for yeast. COMMGEN increases coherence with the actual biological system while maintaining predictive power. This balance is of utmost importance for the usability and reliability of GSMs to elucidate cell function interactions.

As demonstrated by our case study for *P*. *putida*, we argue that (semi-) automatically generated consensus models provide the basis for additional improvements due to their comprehensiveness and standardized naming system. Gap-filling methods [2,39] may be able to close gaps that are not apparent in the IGSMs. One can use existing methods [2,40] to re-evaluate reaction directionalities, especially for reactions that differed in the IGSMs. Compartment assignment methods [41] can resolve remaining compartmentalization issues and optimization methods [34,42] may alter the model to increase its predictive ability. Finally, a good consensus model is a solid foundation for new models by providing a basis for GSMs of similar organisms, and via its integration into multi-scale whole-cell or tissue models [36].

More generally, the systematic integration of heterogeneous information is an essentially unsolved challenge in (post-)genomic biology. For metabolism, consensus GSMs are formalized means for complementing incomplete information, and for identifying and addressing errors through the comparison of independently generated GSMs for the same organism. COMMGEN automatically identifies and semi-automatically resolves widespread and highly interlinked inconsistencies between initial GSMs, thereby moving beyond existing approaches for manual and computer-aided consensus model generation. It can therefore facilitate the construction of new models by comparing and combining information from automatic model construction tools such as the modelSEED [43] and manual model construction efforts, and facilitate GSM updates using a reference—both tasks are analogous to consensus GSM generation.

While we focus here on the reconciliation of multiple GSMs for the same species, we argue that COMMGEN’s methods and standardization are more widely applicable. The identification of similar, yet distinct, biochemical entities can help to compare metabolic capabilities of organisms in detail via their GSMs, or even to compare entire pathway databases. However, dealing with different species will require new, systematic preprocessing steps to map gene sets in different organisms functionally to each other (e.g., via orthology or enzyme classification numbers), which is a topic of future research. In addition, COMMGEN’s methods for identifying redundancies and hierarchical relationships in networks can be used to further advance standardization of terms and ontologies. We therefore expect COMMGEN to be of substantial aid in future integration of knowledge for metabolic networks, to greatly accelerate model-building processes and to thereby improve subsequent high-throughput model-based network analyses. Although COMMGEN will not directly address the domain-specific problems, these capabilities will lay a solid foundation for the systematic, genome-scale comparison of metabolic spaces within and across genera and will have substantial impact for large-scale evolutionary analyses, design of microbial communities, and understanding of host-microbe (pathogen, microbiome) interactions.

## Methods

### Genome-scale metabolic models

iJN746 and iJP962 were requested from and received by email from the first authors of the corresponding papers. GSMN-TB was downloaded from http://sysbio3.fhms.surrey.ac.uk/. iNJ661 was obtained from the supplementary files of the corresponding paper. The remaining models were taken from the model repository at www.metanetx.org. See S1 Dataset for details.

### Evaluation of model performance

For comparison to experimental data, the models were loaded into the COBRA toolbox [44]. The bounds of the boundary reactions were adjusted based on the medium composition and, where necessary, additional flexibility was provided to individual models. Gene knockout strains were simulated by removing the reactions requiring the encoded protein. To discriminate growth from no growth for wild type strains a default cut-off value (10^−6^) was used whereas a minimal relative growth rate (30%) to the wild type was used for mutant strains. See S3 Protocol for details.

### Matching metabolites based on network context

In a metabolic network, reaction nodes are only connected to the metabolite and gene nodes that are involved in the corresponding reaction. Similarly, metabolite and gene nodes are only connected to reaction nodes. However, reaction nodes are not informative for the identity of metabolites as two metabolites representing the same chemical compound are non-overlapping in their connected reaction nodes. Therefore, we characterize metabolites by the other metabolite and gene nodes that are connected to the same reactions. We use this information to quantify how similar metabolites from different models are based on their network context. These similarity scores are then compared to the scores of metabolites that are known to match because they are present in both models: pairs of metabolites that score comparable to these shared metabolites may consist of functionally equivalent chemical compounds. We use a user-defined percentile of shared metabolite scores as a threshold to identify similar metabolites. The method is described in the following:

- We create a Boolean metabolite-to-metabolite matrix M_m_ (m x m) where a 1 indicates that the two metabolites share a reaction.
- We create a Boolean gene-to-metabolite matrix M_g_ (g x m) where a 1 indicates that the metabolite and gene share a reaction.
- We create an attribute matrix M_a_ ((m + g) x m) by vertically concatenating M_m_ and M_g_.
- We normalize M_a_ by dividing each row by its sum such that the numbers in each row sum up to 1. Thereby, the values in M_a_ reflect both that a metabolite is connected to a metabolite or gene and how rare (defining) this connection is.
- We discard rows from M_a_ that correspond to metabolites and genes that are not included in both models for these cannot aid in the identification of common metabolites between the models.
- We discard the columns from M_a_ that correspond to metabolites that are identified to be the same in both GSMs.
- We create a scoring matrix M_s_ (m x m) where the number at position i,j corresponds to the Pearson’s correlation coefficient between columns i and j of M_a_.
- We distinguish between similar and non-similar metabolites in M_s_ using a minimal score. The minimal score equals a user-defined percentile of scores for metabolites that are present in both models.

### Identification of lumped reactions

A lumped reaction is an artificial reaction that represents the net effect of multiple individual reactions. Therefore, if the lumped and non-lumped representations carry flux in opposite directions, steady state is maintained as they cancel each other out. We use this property to identify lumped reactions by linear programming. The method is described in the following:

- We determine the directionality for each reaction as forward, backward, or reversible.
- We transform each reaction such that it only runs in the forward direction; backward reactions are reversed and reversible reactions are split into two reactions.
- We update the stoichiometric matrix S (m x r) accordingly.
- We remove the boundary reactions from S as these reflect exchanges of metabolites between the organism and the medium.
- We define the linear programming (LP) problem:
  $$\text{max}\{c^{\prime }x\}$$
  s.t.
  $$S_{irr}x=b$$
  lb ≤x≤ub
- We initiate the variables of the LP problem
  **c**: Vector (1 x r) containing the objective coefficient for each reaction. We set each value to -1 to penalize flux through each reaction; this ensures that the total flux in the network is minimized.
  **lb**: Vector (1 x r) containing the lower bounds of each reaction. As all reactions are forward reactions, every value is set to 0.
  **ub**: Vector (1 x r) containing the upper bounds of each reaction. As all reactions are forward reactions, every value is set to 1000.
  **b**: Vector (m x 1) containing the desired accumulation or dissipation of each metabolite. Each value in this vector is set to 0 to ensure a steady-state flux distribution.
- We select a reaction LR with index i_LR_ to be considered as a lumped reaction. We set:
  **c**(i_LR_) = -1000
  **lb**(i_LR_) = -1.
  LR is thus now allowed to carry flux in the backward direction, which results in a positive contribution to the objective value.
- We run the LP problem as defined under step v.
  The LP problem returns a flux distribution **x** that either only contains zeros (no non-lumped representation available), or contains a flux distribution such that the flux through LR is maximized in the reverse direction while having a minimal flux through the rest of the network. In the first case, we skip steps ix and x. In the latter case, we identified a set NL_1_ of corresponding non-lumped reactions.
- We save the set NL_1_ for future reference.
- We modify the LP problem such that any alternative sets NL_x_ may be identified.
  **c**(NL_1_) = 3 x **c**(NL_1_)
  This effectively further penalizes flux through the reactions of NL_1_ such that it becomes more ‘rewarding’ to use other reactions.
- We repeat steps viii-x (replace NL_1_ by NL_2_, NL_3_, …) until:
  - No non-zero solution to the problem exists, or
  - The number of reactions in NL_x_ exceeds a user-defined threshold (default: 5), or
  - There is a recurring set NL_x_.
- We filter the different sets NL_x_ such that only sets remain that overlap to a pre-defined extent in gene associations with LR.
- We repeat steps v-xi such that we obtain sets NL for each reaction in the model.

### Identification of alternative transport

Alternative transport reactions result in the transport of a metabolite between two compartments with a different net reaction. We identify metabolites with alternative transport reactions one metabolite at a time. If a metabolite is present in two or more compartments, we identify all transport reactions for this metabolite by selecting reactions where the metabolite is on both sides of the equation. If two of these reactions transport the metabolite between the same two compartments, these reactions are alternative transport reactions.

### Identification of invalid transport

Invalid transport reactions are reactions that transport metabolites between two unconnected compartments. We identify these by forming a list of all compartments that are directly connected through transport reactions in the IGSMs and asking the user to indicate if any of these are invalid. For any of the invalid compartment connections, we identify reactions that contain metabolites from both compartments; these reactions are invalid transport reactions.

### Identification of alternative compartmentalization

We create a separate stoichiometric matrix S_cmp_ (m x r) for each compartment. These matrices only contain reactions of which all metabolites are in the same compartment. Columns (reactions) that are identical between these matrices represent identical reactions with an alternative compartmentalization.

### Identification of unknown compartment

In the MnXRef namespace, metabolites with an unclear compartmentalization are placed in the compartment UNK_COMP. For each reaction that contains a metabolite in UNK_COMP, we identify reactions from the other IGSM(s) that involve all metabolites with known compartmentalization similarly to the identification of alternative stoichiometries. These reactions are then filtered for reactions that also involve the metabolite with the unknown compartmentalization.

### Identification of invalid boundary reactions

Boundary (exchange) reactions are artificial reactions that represent the exchange of metabolites with the medium. They only involve a single metabolite, and have no metabolites on the other side of the equation. In some models these reactions are lumped together with transport reactions that import metabolites from the extracellular compartment. After the MnXRef namespace conversion these reactions are still annotated as boundary reactions, and are thus easily identified in COMMGEN by searching for boundary reactions with non-extracellular metabolites.

### Removing a compartment

To combine GSMs with an alternative compartmentalization, it is sometimes most straightforward to remove a compartment ‘RC’ from a GSM and move its reactions to a different target compartment ‘TC’. We defined four categories of reactions in RC, which are treated differently when RC is removed: (i) Reactions that only involve metabolites from RC are moved to TC; (ii) Multi-compartment reactions that transport a metabolite between RC and TC are removed; (iii) Multi-compartment reactions involving RC and TC that involve a chemical conversion are kept, but all metabolites from RC are placed in TC; (iv) Multi-compartment reactions involving RC and a metabolite other than TC are kept, and all metabolites from RC are placed in TC.

### Identification of identical net reactions

Identical net reactions are reactions that involve the same set of metabolites in the same stoichiometries, but they may be defined in opposing directions. Therefore, we create a double stoichiometric matrix S_dbl_ (m x 2r) that contains the normal stoichiometric matrix S (m x r), as well as its negative -S (m x r). We then identify columns (reactions) in S_dbl_ that are identical.

### Identification of alternative stoichiometries

We convert the S (m x r) matrix to a Boolean (0/1) representation S_log_ (m x r). We then identify columns in S_log_ that are identical; these correspond to reactions involving the same metabolites, but in different stoichiometries.

### Identification of alternative redox pairs

GSMs often differ in their involvement of redox pairs in any particular reaction. The first step in identifying these inconsistencies is the creation of a list of redox pairs. COMMGEN comes with a list of commonly used redox pairs in the MnXRef namespace, and this list can be expanded by the user. COMMGEN can suggest expansions for this list by selecting metabolite pairs that co-occur frequently (≥ 80% of reactions). We identify reactions that are identical except for their redox pairs by expanding the stoichiometric matrix S (m x r) to S_rdx_ (m+1 x r) by adding an artificial metabolite ‘redox pair’. Then, for each reaction that involves a redox pair, we put the stoichiometric coefficients of the redox metabolites in S_rdx_ to ‘0’, and add a ‘1’ in the ‘redox pair’ row instead. We then use the same approach as for the identification of alternative stoichiometries to identify reactions that only differ in stoichiometries and redox pairs.

### Identification of nested reactions

We convert the S (m x r) matrix to a Boolean (0/1) representation S_log_ (m x r). For each column (reaction) we then identify other columns that contain nonzero elements on each row where the respective column has a nonzero element. These sets of columns (reactions) are potentially nested reactions. We then confirm these sets by detecting sets where two or more metabolites that are on the same side of the equation for one reaction, are on the same side of the equation for the other reaction.

### Identification of similar reactions

Similar reactions are reactions from different IGSMs that share a predefined number of genes, substrates and products. We identify similar reactions by constructing three sets of pairs of reactions: (i) reactions that originate from different IGSMs, (ii) reactions that share the required number of substrates and products, and (iii) reactions that share the required number of genes. All combinations of two reactions in each of these three sets are considered similar reactions.

### Implementation and simulation

All computational simulations and analyses were performed using MATLAB [45]. Gurobi [46] was used as linear programming solver for flux balance analysis.

### Namespace conversion

COMMGEN uploads SBML files to MetaNetX.org [47], where the namespace conversion into MnXRef [31] is performed, and downloads the resulting model. Because errors may be introduced at this stage (incorrect namespace conversion of individual metabolites) the mapping is presented to the user who can reject incorrect matches. See S4 Protocol for details.

### File formats and accessibility

The COMMGEN version used for this paper is freely available as MATLAB code as S6 Protocol. A current version of COMMGEN can be found at https://gitlab.com/Rubenvanheck/COMMGEN.

## Supporting Information

S1 Dataset Models. This file contains the original models, the input models, the BCMs, and the RCMs for COMMGEN as well as an overview of the changes made between original and input models.
(ZIP)

S1 Protocol Automatic RCM creation. This file contains the code that was used in order to obtain the data for Fig 3a–3c.
(ZIP)

S2 Protocol Effect of COMMGEN on gene rules. Upon the merging of reactions differing in gene rules a choice has to be made in how the final gene rule looks. This file shows how the consensus procedure as applied for this study affects the use of ‘OR’ and ‘AND’ operators.
(ZIP)

S3 Protocol Growth phenotypes. This file contains the scripts and reference data for the prediction of growth and no-growth phenotypes and subsequent creation of Fig 5a.
(ZIP)

S4 Protocol Example scripts. This file contains two example scripts of how to start with COMMGEN.
(ZIP)

S5 Protocol Matching of metabolites between models. This file contains the code that was used in order to obtain the ROC curve in Fig 2c.
(ZIP)

S6 Protocol COMMGEN. This file contains the code for COMMGEN and is recommended to use when running scripts from other additional files. However, we recommend obtaining the current version of COMMGEN from https://gitlab.com/Rubenvanheck/COMMGEN.
(ZIP)
