# Supplementary material for: Efficient Reconstruction of Predictive Consensus Metabolic Network Models
Source: PLoS Comput Biol. 2016 Aug 26;12(8):e1005085. doi: 10.1371/journal.pcbi.1005085 (PMC5001716; doi:10.1371/journal.pcbi.1005085)
Supplement: S2 Protocol — Upon the merging of reactions differing in gene rules a choice has to be made in how the final gene rule looks. This file shows how the consensus procedure as applied for this study affects the use of ‘OR’ and ‘AND’ operators. (ZIP) [file pcbi.1005085.s003.zip › S2-GpR/S2_GPR.docx]

Supplementary data 1 – GpR

COMMGEN matches reactions that are alternative representations of the same biological process in different genome-scale metabolic models (GSMs). These reactions can differ in the associated gene-protein-reaction rules (GPR). The differences can be manually inspected on a case-by-case basis, or can be automatically resolved according to pre-defined settings.

The models that were generated in this study were created using pre-defined settings. By default, different gene rules were combined using a Boolean ‘OR’ statement. However, if one of the GSMs indicated that a set of genes corresponds to a protein complex, this information was maintained; Boolean ‘AND’ statements were given precedence over ‘OR’ statements.

See Table 1 for examples.

Table 1 Examples of gene rule merging

| Original 1 | Original 2 | Final |
| --- | --- | --- |
| A | B | A OR B |
| A AND B | B AND C | (A AND B) OR (B AND C) |
| A OR B | A AND B | A AND B |
| A | (A AND B AND C) OR D | (A AND B AND C) OR D |
